# Organizational determinants of missed nursing care in surgical

**DOI:** 10.1007/s11845-026-04306-1

**Published:** 2026-04-10

**Authors:** Hamide Şişman, Şeyma Yurtseven, Dudu Alptekin, Derya Gezer

**Affiliations:** 1 Department of Nursing, Selcuk University Aksehir Kadir Yallagoz Health College, Konya, Turkey; 2 Department of Çukurova University Faculty of Medicine, Balcalı Hospital Adana, Adana, Turkey; 3 Departman of Nursing, İzmir Katip Celebi University, İzmir, Turkey; 4 Department of Nursing, Faculty of Health Sciences, Tarsus University, Tarsus, Mersin Turkey

**Keywords:** Missed nursing care, Nursing staff, Patient safety, Surgical nursing

## Abstract

**Purpose:**

This study examined the prevalence and systemic causes of missed nursing care (MNC) in surgical units and investigated the relationship between missed care and nurses’ professional satisfaction to support global patient safety strategies.

**Methods:**

This descriptive, cross-sectional study included 107 registered surgical nurses at a university hospital in 2024. The validated MISSCARE Questionnaire was utilized, demonstrating high reliability (Cronbach’s alpha = 0.915). Data were analyzed using descriptive and inferential statistics.

**Results:**

Primary causes of MNC were insufficient workforce (87.9%) and equipment shortages (75.7%). Critical safety omissions were prevalent; 60.7% of nurses reported that timely medication administration and 57.9% reported that regular vital sign monitoring were rarely achieved. Notably, 57.9% of nurses liked their profession only at a “moderate” level, a sentiment linked to the 87.9% staffing shortage preventing quality care. Nurses who "did not like their job at all" had significantly higher MNC scores (*p* = 0.001), establishing that understaffed environments impair both professional satisfaction and patient safety.

**Conclusions:**

MNC in surgical units is a systemic problem caused by workforce and logistical inadequacies, threatening patient safety. Job satisfaction is a direct organizational predictor of MNC (*p* < 0.001), suggesting that investing in positive work environments is crucial for clinical quality. We advocate for the global implementation of surgery-specific nurse-patient ratios.

## Introductıon

Inadequate nursing care is a growing problem in global healthcare systems, posing a serious threat to patient safety. Missed nursing care as "nursing care activities that nurses know are necessary but cannot perform due to various barriers," is a significant factor that negatively impacts patient outcomes, particularly in surgical clinics. The concept of "missed nursing care" is closely related to "incomplete nursing care," but there are some nuances between these two concepts in the literature. Uncompleted maintenance generally refers to tasks not being completed on time or being done incompletely, while missed maintenance more strongly emphasizes the conscious or unconscious complete omission of these tasks (Griffiths, et al., 2024). This condition is particularly prevalent in surgical clinics and can result in adverse outcomes for patients during the postoperative period [5]. The most common types of missed care in surgical patients include infection control, timely pain assessment, ensuring mobility, and inadequate patient education [13]. These deficiencies cause patients’ recovery to be delayed, hospital costs to increase, and patient satisfaction to decline (Griffiths et al., 2024).

Understanding the underlying causes of missed nursing care is vital for developing effective solutions to this problem. Systematic literature reviews indicate that inadequate nurse staffing, unfavourable working conditions, communication issues, lack of leadership support, and nurse burnout are the most significant factors contributing to this situation (Bragadóttir et al., 2017). The increased workload and emotional pressure, especially after the COVID-19 pandemic, have led to a further increase in missed care cases. The meta-analysis by Zhang et al. [23] reveals that missed nursing care is directly associated with serious adverse outcomes for patients, including infection, medication errors, falls, and even death. Additionally, it is stated that this situation also negatively affects nurses’ job satisfaction [23]. Various compilations highlight that deficiencies in supplies, materials, human resources, and communication are the main causes of missed care, with high workload and the nursing shortage being key factors. These findings clearly show that missed nursing care is a global public health and management issue, leading to significant economic burdens for health systems [3]. Papathanasiou et al.'s [18] umbrella review of 62 studies noted that missed nursing care in surgical units was most common in the areas of "pain management," "mobilization," and "infection control," but noted a lack of local data to support these findings [18].

While numerous studies exist in the literature on the causes of nursing care shortages, meta-analyses and systematic reviews have identified inadequate staffing, adverse working conditions, and burnout as significant contributing factors to missed nursing care. However, systemic barriers (personnel, equipment), such as high-efficiency large units, rapid deployment, and provider job satisfaction, are prevalent and need to be examined to address critical areas of primary care (nutrition) [18, 23]). In the systematic review conducted by Griffiths et al. (2024), the main reasons were listed as insufficient staff, lack of materials, and communication problems (Griffiths et al., 2024). However, the specific context of these findings in surgical units has not been sufficiently clarified. Considering the increased workload and staff shortages, especially in the post-COVID-19 period, it is believed that the findings of this study will offer significant implications for both clinical practices and health policies. Our study aims to develop evidence-based solutions to improve the quality of surgical care, in line with the World Health Organization’s [21] “Safe Surgery” guidelines (WHO, 2021).

## Methods

### Study design

A descriptive, cross-sectional study design was adopted for the study design. This specific hospital was chosen due to its high volume of surgical patients and its role as a regional trauma center. Research data were collected by researchers between July-August 2024.

### Sample

The study population consisted of 118 surgical nurses, and the research was completed with 107 participants who met the inclusion criteria. This indicates a **participation rate of 90.6%**, which underscores the high representativeness of the sample and significantly strengthens the methodological reliability of the study’s findings. This high response rate minimizes potential non-response bias and ensures a comprehensive reflection of the professional environment within the surgical units.

### Data collection

The data collection process was conducted between July-August 2024 through structured face-to-face interviews. To mitigate potential social desirability bias and ensure the highest level of participant confidentiality, a self-administration method was utilized where participants placed their completed questionnaires into sealed envelopes. Each session typically lasted between 10 and 15 min. Data were collected by the researcher using the 'Personal Information Form’ and the 'Missed Nursing Care Scale,' which were developed and refined based on a comprehensive literature review. Prior to data entry, each participant was briefed on the study’s objectives, and their voluntary participation was secured through both verbal and written informed consent.

### Data collection tools

The data were collected by the researcher using the "Personal Information Form" and the "Missed Nursing Care Scale", which were collected in light of the literature.

### The personal information form

The form prepared by the researcher based on the literature review consists of questions (10 questions) that inquire about the nurses’ gender, age, marital status, education level, total years of work experience, years of work experience in the surgical clinic, working hours, job position, level of liking their profession, and level of satisfaction with their clinic (İlaslan & Şişman, 2019; [7]). The survey were collected by the researcher through face-to-face interviews with nurses working in the surgical clinic.

### Missed Nursing Care Scale (MISSCARE)

The MISSCARE Survey, developed by Kalisch and Williams (2009) and adapted into Turkish by Kalisch et al. [15], was used to identify the elements of and reasons for missed nursing care. The scale consists of 41 items in total, divided into two sections. Section A contains 24 items related to the elements of nursing care, scored on a 5-point Likert scale (1 = never missed to 5 = always missed). Section B contains 17 items concerning the reasons for missed care, scored on a 4-point Likert scale (1 = not a reason to 4 = significant reason).

For the purposes of this study, a total MISSCARE score was calculated by summing the responses across all 41 items. Consequently, the theoretical score range is between 41 and 188, where higher scores indicate a greater frequency of and more significant reasons for missed nursing care. Defining this range provides a clearer context for interpreting the mean scores obtained in the findings. In this study, the workforce sub-dimension was 0.729, the materials sub-dimension was 0.601, the communication/teamwork sub-dimension was 0.863, the Cronbach’s alpha value for the first part of the scale was 0.915, and the total score for the second part was 0.885.

### Data analysis

The data collected in the study were analyzed using SPSS (Statistical Package for Social Sciences) for Windows version 25.0. Descriptive statistical methods, including the number, percentage, mean, and standard deviation, were employed to evaluate the data. The Kolmogorov–Smirnov test was used to assess the suitability of variables for normal distribution. One-way ANOVA test was used to determine the difference between three or more independent groups with a normal distribution. A Bonferroni correction was applied in Post-Hoc analyses to determine which group caused the significant difference. Results were evaluated at a 95% confidence interval and a significance level of *p* < 0.05.

### Ethical considerations

Ethical approval for the study was obtained from the Çukurova University Non-Interventional Clinical Research Ethics Committee (Date: June 14, 2024; Decision No: 145/2). Additionally, official institutional permission for the study was obtained from the relevant healthcare institution (Date: July 20, 2024; Number: E-59565534-209.01-1057883). All participants provided written informed consent before inclusion in the study, and the study was conducted in accordance with the principles of the Declaration of Helsinki.

## Results

The findings obtained from 107 surgical nurses who participated in the study are presented under three main headings, referencing Tables 1, 2, and 3: demographic characteristics, reasons for missed nursing care (MNC), and the effect of professional satisfaction level on missed care scores.

**Table 1:** Analysis of demographic data of participants (*n *= 107)

Variables	n (number)	% (percentel)
Gender		
Female	86	80.4
Male	21	19.6
Marital status		
Married	77	72
Single	30	28
Education		
Health vocational high school	23	21.5
Associate degree	19	17.8
Bachelor’s degree	39	36.4
Master’s degree/Doctorate	26	24.3
Working position		
Clinical nurse manager	9	8.4
Clinical nurse	98	91.6
Working schedule		
Daytime only (08:00-16:00)	55	51.4
Day+Night (08:00-16:00/16:00)	47	43.9
Only on-call (16:00-08:00)	5	4.7
Level of job liking		
Not at all	10	9.3
A little	14	13.1
Moderate	62	57.9
A lot	21	19.6
Level of satisfaction with the clinic wherework		
Not at all	5	4.7
A little	7	6.5
Moderate	56	52.3
A lot	39	36.4
Variables	Mean±SD	Min-Max
Age	34±7	25-56
Total years of practice	12±8	3-38
Years of practice in a surgical clinic	9±8	2-38
Total unmet care needs scale score	78±13.7	24-99
Reasons for unaffordable nursing care services		
Labor resources	15±2.2	8-16
Communication/Teamwork	27±5.2	15-33
Material resources	11±1.8	6-12

* Data are expressed as number (n), frequency (%), mean (average), minimum-maximum (Min-Max)

**Table 2:** Evaluation of factors causing missed nursing care

Why sub dimension	Ort±SD	Min-Max	Highest frequency reason (I completely/Frequently agree)	Frequency (%)
Workforce resources	12.5±2.4	8-16	Insufficient number of staff	87.9
Material resources	8.1±1.9	6-12	Inadequate or defective equipment/materials	75.7
Communication/Teamwork	23±4.7	15-33	Failure of other healthcare personnel to provide necessary care	52.3

Since the Cronbach’s Alpha value of the Material Resources sub-dimension used in this table was found to be low at 0.601, these findings should be interpreted with caution

**Table 3:** Effect of professional satisfaction level on missed nursing care points (*n *= 107)

Variable group	Number (n)	Mean.±SD	Statistical analysis*	Significant differences
Status of loving the profession				
I don’t like it at all (1)	10	51.0±5.2	F (3.103)=21.5, *p *= 0.001*	1>2>3>4
I like it less (2)	14	48.5±6.1		
Intermediate (3)	62	39.2±4.8		
I love it very much (4)	21	35.1±3.9		
Satisfaction level with the clinic worked				
Very dissatisfied (1)	5	53.2±5.5	F (3.103)=15.3, *p *= 0.001*	1>2>3>4
Less satisfied (2)	7	50.9±6.8		
Intermediate (3)	56	41.8±7.1		
Very satisfied (4)	39	37.5±5.9		

F: Variance analysis statistical value. p: Statistical significance level. *SD* Standard deviation; *Avg.* Average; *n* Number of participants in the group

The majority of nurses participating in the study were female (*n* = 86, 80.4%) and married (*n* = 77, 72%). When educational levels were examined, the largest group consisted of Bachelor’s Degree graduates (*n* = 39, 36.4%), followed by those with a Master’s or Doctoral degree (*n* = 26, 24.3%). A large proportion of participants worked in the Clinical Nurse position (*n* = 98, 91.6%) and primarily worked on the Daytime Schedule (*n* = 55, 51.4%). The mean age of the participants was 34 ± 7 years, and the mean total professional experience was 12 ± 8 years. Regarding their level of job liking, 57.9% (*n* = 62) of the nurses stated they liked their profession at a “Moderate” level. Only 19.6% (*n* = 21) stated they liked it “A Lot”, while 9.3% (*n* = 10) stated they liked it "Not at All". In terms of satisfaction with their current clinic, the highest rate was also in the “Moderate” satisfaction group (*n* = 56, 52.3%). The mean total score for the Missed Care Needs Scale was found to be 78 ± 13.7 (Table 1).

The factors causing missed nursing care were analyzed across the three main sub-dimensions of the MISSCARE Questionnaire using descriptive statistics. The sub-dimension with the highest mean score was the Workforce Resources sub-dimension (Mea*n* = 12.5 ± 2.4). In this subscale, 87.9% of nurses reported "Insufficient number of staff" as the reason for missed care with the highest frequency. The sub-dimension with the second-highest mean score was the Material Resources sub-dimension (Mea*n* = 8.1 ± 1.9). Regarding material resources, the data **suggests a clear trend** toward logistical challenges, with 75.7% of nurses citing 'Inadequate or defective equipment/materials’ as a primary reason for missed care. However, as the Cronbach’s Alpha value for this sub-dimension (0.601) indicates borderline internal consistency, these specific findings should be interpreted with caution as indicative trends rather than definitive metrics. The sub-dimension with the lowest mean score was the Communication/Teamwork sub-dimension (Mea*n* = 23.0 ± 4.7). In this sub-dimension, 52.3% of nurses stated that the "Failure of other healthcare personnel to provide necessary care" was a reason for experiencing MNC (Table 2).

A statistically significant difference was found between the nurses’ Status of Loving the Profession and their total Missed Care scores (F(3,103) = 21.5, *p* = 0.001). The highest mean MNC score was observed in the group that stated they "Don’t Like It at All" (51.0 ± 5.2), while the lowest score was in the group that stated they "Love It Very Much" (35.1 ± 3.9). Bonferroni Post-Hoc analysis showed that the MNC score of the "Don’t Like It at All" (1) group was significantly higher than the scores of the “Intermediate” (3) and “A Lot” (4) groups. Additionally, the score of the "Like It Less" (2) group was significantly higher than the score of the "Love It Very Much" (4) group. Similarly, a statistically significant difference was found between the Level of Satisfaction with the Clinic and the total Missed Care scores (F(3,103) = 15.3, *p* = 0.001). The highest mean MNC score was observed in the "Not at All" satisfied group (53.2 ± 5.5), while the lowest score was found in the “A Lot” satisfied group (37.5 ± 5.9). Bonferroni post hoc tests confirmed that the MNC score of the "Not Satisfied At All" (1) group was significantly higher than the scores of the "Somewhat" (2), "Moderate" (3), and "Very Satisfied" (4) groups (Table 3).

## Discussion

Our findings provide robust, single-centre data on the prevalence and organizational predictors of MNC in surgical units, addressing a critical knowledge gap in global patient safety science. The extent of these care omissions questions the ethical and clinical implications of the hierarchical prioritization of nursing care by showing that even meeting basic human needs, such as serving a hot meal to the patient (routinely omitted) and ensuring mobilization, are frequently neglected..addressing a critical knowledge gap in global patient safety science. Surgical units are characterized by high patient severity and rapid turnover, requiring constant vigilance and timely interventions [1]. In this study, the identified 87.9% staffing shortage creates a critical safety gap where essential nursing tasks, such as patient mobilization, are frequently bypassed [18]. It is imperative to emphasize that missed mobilization in a surgical patient is not 'just a deficiency’ in care,it is a life-threatening risk [23]. Failure to mobilize patients significantly increases the risk of severe surgical complications, including post-operative infections and deep vein thrombosis, leading to prolonged hospital stays and increased mortality [1],WHO, 2021). This high-risk environment, where nurses are structurally prevented from ensuring patient safety due to understaffing, directly impacts their professional attachment. The finding that 57.9% of nurses like their profession only at a ‘moderate’ level is a reflection of this chronic moral distress [20]. When nurses cannot prevent life-threatening outcomes due to the 87.9% workforce deficit, their professional satisfaction erodes, transforming a rewarding clinical practice into a stressful management of potential complications [18, 20]. Surgical units are characterized by high patient severity and rapid turnover, requiring constant vigilance and timely interventions. In this study, the identified 87.9% staffing shortage creates a critical safety gap where essential nursing tasks, such as patient mobilization, are frequently bypassed. It is imperative to emphasize that missed mobilization in a surgical patient is not 'just a deficiency’ in care,it is a life-threatening risk. These findings are fully consistent with Kalisch’s MISSCARE theory [14], which points out that nurses are forced to deliberately bypass essential components of holistic care while focusing on critical medical tasks. Neglecting basic nutrition and mobilization increases surgical patients’ vulnerability to infections, prolongs recovery times, and increases hospital readmission rates, imposing high costs on healthcare systems [18]. This suggests that nursing science urgently needs to re-emphasize the central and vital role of fundamental acts of care in patient survival. Moreover, significant deficiencies in the timely administration of medications (with 60.7% of nurses reporting this is rarely achieved) and regular vital sign monitoring (reported as seldom performed by 57.9% of nurses) prove that the World Health Organization (WHO) 'Global Patient Safety Action Plan’ goals (WHO, 2021) are critically endangered by systematic barriers in surgical units.

Our findings confirm the global scope and urgency of the current nursing workforce crisis in surgical units, with understaffing cited by 87.9% of respondents as the most important cause of MNC. This structural deficiency directly correlates with the finding that 57.9% of nurses like their profession only at a ‘moderate’ level, suggesting that chronic staffing shortages act as a primary inhibitor of professional fulfillment. When 87.9% of the workforce operates under constant resource pressure, the resulting moral distress stemming from the inability to provide the high-quality care they were trained for likely prevents nurses from moving beyond a ‘moderate’ attachment to their roles. This environment transforms nursing from a vocation into a high-stress management of omissions, directly linking the 87.9% staff shortage to the erosion of professional enthusiasm. Surgical units are particularly sensitive environments due to high patient severity and rapid changeover requirements. In parallel with the studies of Aiken et al. [1], such a high staff shortage rate shows that MNC should be considered as a managerial failure and structural violence against nurses. Given the complexity of surgical care, enacting and implementing evidence-based, surgery-specific nurse-patient ratios internationally is a critical policy imperative.

The high reporting rate of equipment and material shortages (75.7%) emphasizes that MNC is not only a human-caused problem but also a result of profound inadequacies in hospital logistics and supply chain management. This indicates an urgent need to revise resource allocation policies to eliminate systemic barriers that prevent nurses from fully fulfilling their professional roles. However, given that the Cronbach’s Alpha value of the material resources sub-dimension was found to be at the borderline reliability of 0.601, these findings must be interpreted with caution, as the low reliability of this specific sub-scale limits the generalizability of the reported high shortage rate to other clinical settings.

The most striking finding of the study is the advanced statistical evidence that MNC scores significantly and strongly increase with the decrease in nurses’ level of professional liking and satisfaction with the clinic (*p* = 0.001). This finding proves that nurses’ job satisfaction is not merely an indicator of individual well-being but a direct organizational predictor of patient safety and quality of care. Consistent with the literature [5] that nurses with low job satisfaction are more prone to MNC, our findings provide surgical unit leaders with a critical strategic direction: Investing in job satisfaction is the most cost-effective and sustainable way to reduce missed care actions. This investment should include providing better work environments, leadership that encourages autonomy [16], and adequate rest breaks, as stressed by Hernández et al. [10], to mitigate burnout. Consequently, high professional commitment and satisfaction serve as a cognitive reservoir that allows nurses to set more ethical and comprehensive care priorities even under high workload.

### Limitations and strengths

This study was conducted in the surgical clinics of a single university hospital, and generalization of the findings nationally or internationally is limited. In addition, the Cronbach’s Alpha value of the material resources sub-dimension of the MISSCARE scale has a borderline reliability of 0.601, which requires caution in interpreting the findings regarding this sub-dimension. However, the detailed local data obtained provide a solid basis for international comparative studies to advance nursing science and shed light on global policy reforms.

Beyond addressing the lack of local data, the primary strength of this study lies in its focus on the critical intersection between organizational predictors and postoperative patient safety. While global literature extensively documents nursing shortages, this research emphasizes that job satisfaction is not merely a measure of staff well-being but a fundamental determinant of clinical integrity in surgical settings. By establishing a direct link between professional satisfaction and the completion of vital safety actions such as medication timing and vital sign monitoring this study underscores that fostering a positive nursing work environment is as vital as logistical resource management in preventing adverse postoperative outcomes.

## Conclusıon

This study conclusively demonstrates that Missed Nursing Care (MNC) in surgical units is a deep-rooted systemic failure resulting from critical workforce deficiencies (cited by 87.9% of nurses) and material resource limitations. The primary finding is the robust, statistically significant inverse relationship between nurses’ professional satisfaction levels and their total missed care scores (*p* = 0.001). This evidence firmly establishes job satisfaction not merely as an indicator of individual well-being, but as a direct, potent organizational predictor of patient safety and quality of care.

Based on these findings, the following urgent policy and management actions are warranted:

- **Policy and Regulatory Imperative:** International health authorities (particularly the WHO) must be alerted to the threat MNC poses to “Safe Surgery” goals, evidenced by critical omissions in essential tasks such as timely medication administration (reported as rarely/never achieved by 60.7% of nurses) and regular vital sign monitoring (57.9%). There is an urgent necessity for the global, swift implementation of evidence-based, surgery-specific nurse-to-patient staffing ratios to ensure the integrity of fundamental care.
- **Managerial Action:** Hospital administrations must acknowledge that high rates of staffing and equipment shortages represent structural and managerial failures rather than individual nurse shortcomings. Investments aimed at boosting job satisfaction (including fostering autonomous leadership and ensuring adequate rest periods) should be prioritized as the most cost-effective and sustainable institutional strategy for substantially reducing the incidence of missed care actions.

## Relevance for clınıcal practıce

Based on the findings of this study, the following implications are highly relevant for clinical practice, nursing management, and patient safety in surgical units:

- **Prioritize Staffing and Logistics:** Clinical managers should recognize that the high incidence of Missed Nursing Care (MNC) appears to be primarily a structural issue, often stemming from insufficient staffing (87.9%) and material shortages (75.7%). Regular monitoring of nurse-to-patient ratios and streamlining supply chain management, particularly during peak surgical recovery periods, may serve as effective interventions.
- **Invest in Nurse Job Satisfaction as a Safety Measure:** The strong inverse relationship between job satisfaction and MNC scores indicates that leadership could benefit from viewing investment in nurse well-being as a direct patient safety strategy. Interventions focused on fostering supportive work environments, promoting nurse autonomy, and ensuring adequate rest periods are recommended to mitigate the pressures of high-stress surgical settings.
- **Systemic Risk Mitigation for Essential Tasks:** The frequent omission of critical safety actions, such as timely medication administration and vital sign monitoring, suggests a need for unit-specific safety protocols that remain mandatory even during high-workload periods. Implementing technology or simplified processes may help ensure these non-negotiable tasks are completed regardless of current staffing levels.
- **Re-emphasizing Fundamental Care:** Nursing education and unit policies should re-emphasize the clinical criticality of basic care elements, such as mobilization, patient nutrition, and hygiene. Regular audits by managers may help ensure these fundamental holistic care components, which directly impact recovery and infection risk, are consistently prioritized despite workload pressures.

## Data Availability

The datasets used and/or analyzed during the current study are available from the corresponding author upon reasonable request.
